# Preferences for mental health treatment for post-partum depression among new mothers

**DOI:** 10.1186/s13584-019-0354-0

**Published:** 2019-12-05

**Authors:** Meital Simhi, Orly Sarid, Julie Cwikel

**Affiliations:** Spitzer Department of Social Work and the Center for Women’s Health Studies and Promotion, Ben Gurion University of the Negev, POB 653, 84105 Beer Sheva, Israel

**Keywords:** Treatment preferences, Postpartum depression, Health policy, Mothers, Demography

## Abstract

**Background:**

The prevalence rate of *postpartum depression* (PPD) is 9 to 17% among mothers, with higher rates among low income and immigrant populations. Due to the negative effects of PPD symptoms on both the mother and baby, treating mothers with depression symptoms is of great importance. This study examined treatment preferences for PPD among Israeli mothers with and without PPD symptoms, specifically focusing on treatment centers, type of professional and mode of treatment, to help develop relevant policies to promote the health of mothers by reaching a deeper understanding of their preferences.

**Methods:**

1000 mothers who attended *Maternal Child Health Clinics* (MCHCs) in Israel for their infant’s first medical exam participated in a cross-sectional survey.

**Results:**

In this sample, 8.4% of the participants suffered from PPD. Mothers with PPD compared to those without symptoms had lower economic status, were more likely to be single, to be first-time mothers, have an unemployed partner and to have immigrated to Israel. Mothers with PPD preferred private mental health practice and community treatment centers by mental healthcare professionals. They also preferred group interventions and personal psychotherapy rather than technology-based interventions.

**Conclusions:**

The study findings support the formulation of mother-sensitive health policies based on understanding mothers’ preferences, and thus, help prepare treatment alternatives that will suit different groups of mothers with PPD, for the benefit of mothers, newborns, and families. Disseminating the results of this study among professionals as part of professional training, can promote appropriate treatment facilities and modes of care for mothers with PPD.

## Background

PPD is characterized by an inability to experience pleasure, anxiety symptoms, panic attacks, spontaneous crying, depressed mood, and sometimes accompanied by suicidal thoughts following the birth of an infant [1]. Epidemiological studies show that the prevalence of PPD among the general population in Western countries ranges between 9 and 17% [2, 3]. Risk factors identified from earlier studies include motherhood at a young age [4, 5], low levels of education and SES [6, 7]. A higher prevalence of PPD symptoms was reported among ethnic minorities and immigrant populations [8, 9].

Women who experience PPD often show signs of self-neglect and engage in risk-related behaviors such as the excessive consumption of alcohol, cigarettes, and illegal substances [5]. The newborn and the other family members can also be affected by the mother’s PPD. Studies have reported that untreated PPD may adversely affect infant–maternal attachment [10], the cognitive and motor development of the infant [10, 11] and was associated with behavioral and learning disorders during childhood and adolescence [12, 13].

Due to the negative effects of PPD symptoms on both the mother and infant, early detection of women with PPD symptoms is of great public health importance. In many Western countries, a routine depression screening is performed among postpartum populations [14]. In Israel, since 2013, nurses routinely assess depressive symptoms during the 26-28th week of pregnancy and 4–9 weeks post-partum in perinatal health visits [15] using a validated translation of the Edinburgh Postnatal Depression Scale (EPDS) [16]. Despite the early detection of mothers with PPD, a large proportion of the mothers are not actually treated for their PPD symptoms [17, 18]. Moreover, results from a study conducted in Australia showed that 20% of women, when screened for perinatal depression and anxiety did not respond honestly. Women who felt uncomfortable reporting PPD symptoms were much more likely to show symptoms of perinatal depression and anxiety [19].

Currently, the treatments offered to women diagnosed with PPD are antidepressants [18]; psychodynamic therapy [20]; interpersonal therapy [21]; and cognitive-behavioral therapies [22], which have all been shown effective in treating PPD.

Previous studies that have examined preferences for treating PPD and showed variations in preferences for type of health care professionals, type of treatment and place of treatment, which differed according to the demographic profile of the mother. For example, a study conducted in the United States showed that compared to younger mothers, older mothers were more likely to seek therapy for their PPD symptoms [23]. Another study found that compared to Caucasian mothers African-American mothers preferred to receive counseling for their PPD symptoms from religious figures in the community and expressed low confidence in medication [24]. Findings from another study showed that educated, married women, with high SES, preferred individual psychotherapy rather than group therapy for their PPD symptoms [25].

Our study population included postpartum women who, according to previous studies [26], were expected to experience mild postpartum “baby blues” (between 60 and 80% of mothers), those who experienced clinical postpartum depression (around 15% in most populations) and those who did not experience any symptoms of depression at all following childbirth. Thus, while it may be difficult to imagine how clinical depression feels, given that the vast majority of new mothers experience some symptoms, we ask them to speculate on what their preferences would be for treatment, given clinical post-partum depression. To the best of our knowledge, no previous studies have compared mothers with and without detected PPD as for their preferences for mental health treatment since it is clear that some women without reported or detected symptoms are still in need of effective and suitable PPD treatment. This was the rationale for the current study.

Our aim was to compare preferences for mental health treatment regarding a variety of mode, profession and place of treatment of mothers with and without PPD. We hypothesized that demographic variables will differ between mothers with and without PPD; we also hypothesized that mothers with PPD compared to those without symptoms would differ as to type of treatments preferences.

### Procedure

A cross sectional study was conducted between December 2014 and August 2015 in eight Maternal and Child Health Clinics (MCHCs) in the center of Israel. The study population included 1000 mothers who attended the MCHCs for the first medical exam of their infant at nine weeks postpartum. Inclusion criteria were Hebrew-speaking mothers who gave birth to a child within the last six months and lived in the region. The study was approved by the research ethics committee of the Ministry of Health, Israel. The sample size was calculated using OpenEpi software for a two-sided significance level of 0.05 and statistical power of 80%. OpenEpi indicated that 900 women were required for the study, which we increased to 1000 as we assumed that approximately 10% would not be fully compatible or would not provide complete data.

We approached mothers who came to MCHCs and presented the purpose of the study. If a mother met the inclusion criteria she was invited to participate. Out of the 1107 eligible mothers 1000 mother (90.2%) agreed to participate, signed informed consent and completed the questionnaire; 7.5% (83) refused to participate and 2.3% [24] had missing data and were not included in the final analysis. Mothers, who were identified as having PPD symptoms, were advised to turn to treatment and were given details about where to access treatment in the vicinity of their residence.

### Study measures

PPD symptoms were assessed using the Edinburgh Postnatal Depression Scale (EPDS) [16]. The questionnaire includes 10 questions, focusing on the feelings of mothers during the perinatal period. Each question is rated on a 4-point Likert scale. The tenth question is about intention to self-harm [27]. The score is the sum of all the statements. In this study, the cut-off point of 10 was selected, similar to previous studies conducted in Israel and in various countries [3, 7, 8]. The internal reliability was good (Cronbach α = 0.82).

Preferences for getting mental health treatment for PPD. The questionnaire was adapted from a previous study to reflect the treatment options available in Israel [25, 28].

- Preferred place of treatment: mothers were asked, “If you felt depressed after birth, to which treatment center would you prefer to go?” Answers were eight different options, for example: MCHCs, Community health clinic (Health Maintenance Organizations, HMOs), psychiatric clinic, or private mental health practice.
- Health Care provider preference: mothers were asked, “If you felt depressed after childbirth, to what extent you would prefer the treatment of the following professionals?” Answers were twelve options, for example: nurses, social workers, or psychiatrist.
- Preference regarding options to receive the therapy: mothers were asked “There are many ways to get treatment. To what extent do you feel that the following options would be suitable for your needs?” Fifteen options were presented, for example: home visits, Skype-based treatment, or group meetings.
- Behavioral intent to access treatment in general was examined with one statement: “If you felt depressed after childbirth, would you go for treatment?” The participant responded on a four point Likert scale ranging from [1] very likely to go for treatment to [4] would not go for treatment at all.

Social-demographic questionnaire: age, ethnicity, years of education, economic status, religion, marital status, number of children, and the employment status of the mother and her husband.

### Sample

In Table 1, we present socio-demographic variables of mothers with and without PPD. Mothers with PPD compared to those without symptoms had statistically significant lower economic status, were more likely to be single, to be first-time mothers, have an unemployed partner and to have immigrated to Israel.

**Table 1:** Demographic variables among women with and without PPD (Percentage (n))

	With PPD	Without PPD	χ2 (df)
*Marital status*			
Single	18.2 (4)	81.8 (18)	6.97* [2]
Married	7.9 (76)	92.1 (883)	
Live with a partner	21.1 (4)	78.9 (15)	
*Number of children*			
First child	12.0 (46)	88.0 (337)	10.90** [1]
Second child and up	6.1 (37)	93.9 (574)	
*Religion status*			
Secular	8.6 (40)	91.4 (427)	0.64 (3)
Traditional	9.2 (25)	90.8 (248)	
Religious	7.5 (12)	92.5 (148)	
Ultra-Orthodox	7.0 (7)	93.0 (93)	
*Immigration*			
Immigrant	12.4 (29)	87.6 (205)	6.33* [1]
Native born	7.2 (55)	92.8 (711)	
*Spouse unemployment*			
Unemployed spouse	16.9 (10)	83.1 (49)	10.17* [1]
Working spouse	7.6 (70)	92.4 (856)	
*Economic status*			
Difficulty	14.9 (28)	85.1 (160)	12.68*** [1]
No difficulty	6.9 (56)	93.1 (756)	
*TOTAL IN THE SAMPLE*	8.4 (84)	91.6 (916)	

*p ≤ 0.05; **p ≤ 0.01; ***p ≤ 0.001

## Data analysis

The data was analyzed using SPSS version 23.0 software. We conducted χ^2^ and student t-tests with demographic, and treatment preference variables among mothers with and without PPD in order to identify salient variables affecting treatment preferences. In the second stage of analysis, we preformed *Exploratory Factor Analysis* (EFA (for the three types of treatment preferences. Variables were included in the analysis, if their loading was .40 and above. We then conducted student t-tests with treatment preference variables after EFA comparing mothers with and without PPD.

## Results

Among our participants 8.4% scored 10 or higher on EPDS. Ten (1%) mothers stated in response to question 10 that they considered hurting themselves.

In Table 2 we present treatment preferences among mothers with PPD compared to those without symptoms. The top three preferred places among mothers with PPD were private mental health practice [1], community health clinic (HMOs) [2], and MCHCs [3]. Mother with PPD ranked community treatment centers in second place compared with women without PPD who ranked it first (t (998) = − 2.55, *p* ≤ 0.05.

**Table 2:** Treatment preferences by PPD status (means, standard deviations, t test)

	With PPD Mean (SD)	Degree of preference	Without PPD Mean (SD)	Degree of preference	t (df = 998)
*Treatment centers*					
Private mental health practice	2.55 (1.26)	1	2.51 (1.26)	2	0.30
Community health clinic (HMOs)	2.38 (1.04)	2	2.70 (1.11)	1	−2.55*
MCHCs	2.26 (1.05)	3	2.49 (1.12)	3	−1.81
Mental health clinics for women in general hospitals	2.10 (1.13)	4	2.09 (1.13)	4	0.11
Community mental health centers	1.84 (1.03)	5	1.78 (0.99)	6	.51
General hospital outpatient clinic	1.75 (0.92)	6	1.81 (0.99)	5	0.13
Psychiatric hospital outpatient clinic	1.60 (0.95)	7	1.52 (0.88)	7	0.84
Inpatient in a psychiatric hospital	1.17 (0.54)	8	1.21 (0.60)	8	−0.59
*Type of professional*					
Psychologist	3.54 (1.49)	1	3.49(1.41)	1	0.37
Alternative therapist (shiatsu, massage, acupuncture)	3.17 (1.52)	2	2.88 (1.52)	3	1.66
Family Physician	2.79 (1.37)	3	2.98 (1.40)	2	−1.16
A family member, friend or co-worker	2.77 (1.40)	4	2.60 (1.45)	4	1.00
Psychiatrist	2.52 (1.51)	5	2.42 (1.43)	8	.58
Art/ Movement/ Music therapist	2.32 (1.46)	6	2.51 (1.52)	6	−1.10
Social Worker	2.30 (1.37)	7	2.55 (1.38)	5	−1.52
Nurse	2.23 (1.21)	8	2.49 (1.36)	7	−1.67
Occupational Therapist	2.17 (1.44)	9	2.30 (1.39)	9	−.82
A volunteer trained for this purpose	2.08 (1.37)	10	2.23 (1.37)	10	−.93
Religious leaders	1.95 (1.43)	11	1.88 (1.35)	11	.47
*Mode of treatment*					
Personal meetings in a private office	2.94 (1.07)	1	3.07 (1.03)	1	−1.10
Home visits	2.59 (1.20)	2	2.61 (1.21)	3	−.11
Group meetings in the community led by a professional	2.48 (1.06)	3	2.27 (1.12)	4	1.65
Personal psychotherapy in a community clinic	2.45 (1.04)	4	2.67 (1.08)	2	−1.84
Group meetings community led by a woman who is not a professional but has experienced similar problems	2.07 (1.06)	5	1.99 (1.06)	5	.60
Internet-based treatment, chatting with another woman in my situation	1.96 (1.05)	6	1.93 (1.04)	6	−.13
Treatment by phone	1.89 (1.04)	7	1.87 (1.06)	7	.13
Medications and treatment	1.80 (1.04)	8	1.87 (1.04)	8	.84
Internet-based treatment, chatting with a professional	1.63 (.95)	9	1.64 (0.94)	11	−.15
Practice on a website with the help and explanation of therapist	1.58 (.94)	10	1.68 (0.96)	10	−92
Using an iPhone application	1.51 (.82)	11	1.70 (1.01)	9	−1.69
Skype with a professional	1.47 (.84)	12	1.53 (.91)	12	−.54
Medication	1.47 (.89)	12	1.39 (0.77)	13	.95
Group meetings on the web with women with the same problems as a self-help group	1.39 (.87)	13	1.35 (0.77)	14	.52
*Would seek treatment in general*	3.04 (1.12)		3.29 (1.00)		−2.12*

*p ≤ 0.05

The preferred professionals were psychologists [1], alternative therapists [2], and family physicians [3]. The top three preferred services among women with PPD were personal meetings in a private office [1], home visits [2], and group meetings in the community under the guidance of a professional [3].

It is important to note that mothers with PPD also were less likely to seek treatment in general (t (998) = − 2.12, *p* ≤ 0.05). No statistically significant differences were found for the other preferences.

In the second stage, of analysis we preformed EFA for all forms of treatment preferences to examine whether the number of components in each preference could be reduced. The analysis showed three main factors in each type of preference. a. *Place of treatment*: community health clinic; psychiatric clinics; and private mental health practice (Appendix 1). b. *Professionals*: professionals in the community; mental health professionals; and paraprofessionals in the community (Appendix 2). c. *Mode of treatment*: personal psychotherapy; technology-mediated interventions; and group interventions (Appendix 3).

In Table 3, we present treatment preferences after the EFA among mothers with PPD compared to those without symptoms. Differences were found between mothers with and without PPD regarding preferences for community treatment centers. Mother with PPD ranked community treatment centers in second place compared with women without PPD who ranked it first (t (998) = − 2.71, *p* ≤ 0.01). No statistically significant differences were found for the other preferences. Mothers with PPD and mothers without PPD preferred private mental health practice (Ranking 1 and 2 respectively) and community treatment centers (Ranking 2 and 1 respectively) and were less likely to want to seek treatment in a psychiatric framework. They also preferred mental health care professionals, group interventions and personal psychotherapy interventions and not technologically mediated interventions.

**Table 3:** Treatment preferences after Exploratory Factor Analysis by PPD status (means, standard deviations, t test)

	PPD mean (SD)	Degree of preference	Without PPD mean (SD)	Degree of preference	t (df = 998)
*Treatment centers*					
Private mental health practice	2.55 (1.26)	1	2.51 (.90)	2	.30
Community treatment centers	2.32 (.84)	2	2.59 (0.90)	1	−2.71**
Psychiatric clinics	1.69 (.68)	3	1.68 (.70)	3	.10
*Type of professional*					
Mental health professionals	3.02 (1.35)	1	2.95 (1.21)	1	.46
Professionals in the community	2.44 (.91)	2	2.67 (1.06)	2	−1.94
Paraprofessionals in the community	2.42 (1.05)	3	2.48 (1.11)	3	−.44
*Mode of treatment*					
Group interventions	2.27 (.96)	1	2.13 (1.01)	2	1.24
Personal psychotherapy	2.25 (.65)	2	2.31 (.63)	1	−.90
Technology mediated interventions	1.62 (.64)	3	1.67 (.67)	3	−.58

**p ≤ 0.01

## Discussion

This study examined comparatively preferences to psychological treatment of mothers with and without PPD. In terms of PPD symptoms, 8.4% of the participants scored 10 or higher. This finding is in accordance with findings of previous studies conducted in Israel among Jewish mothers reporting 9% at cut off point of 10 [8]. While the response rate in our study was high [29], there are still populations that were not represented in this sample, such as Arabic-speaking women whose rates of PPD have been reported as significantly higher than the rest of the population [8].

Our findings showed that women who immigrated to Israel were more likely to suffer from PPD than Israeli-born women. These findings are consistent with previous studies showing immigration as a risk factor for PPD [7, 9]. Women living with a partner had more symptoms of PPD than single or married women. This finding is supported by findings from previous studies [4, 17]. As in other studies we showed that women with poor economic status had more PPD symptoms [4, 30] and preferred to seek treatment less frequently in general [25]. Another finding was that mothers with a first child reported of more symptoms of PPD than mothers with second child and above. The findings in the literature are inconclusive regarding this topic. On the one hand, previous research has shown that women with a first child have more PPD symptoms than women with a second child [31]. However, another study showed a positive correlation between the number of children and PPD [32]. In other studies, however, there was no association between these variables [17, 33].

In the current study, mothers with and without PPD preferred to be treated in private mental health practice and in community treatment centers. They least preferred the option of psychiatric clinics. These findings are consistent with the findings of a previous study conducted in the US. There 90% of mothers preferred to receive primary care in private mental health practice and about 70% preferred to receive primary care in primary care clinics. Only one-fifth of mothers preferred to be treated in mental health care [25]. A possible explanation for our finding is that mothers feared social labeling of mental illness and the stigma associated with being a patient in a psychiatric setting [17, 18, 34]. In Israel, outpatient mental health care services from psychiatric hospitals were transferred in 2015 to HMOs in the community. Among other things, this was done to reduce the labeling of those with mental illness and to facilitate integration into the community of people suffering from mental difficulties. Our study was conducted during the implementation of this policy by the Ministry of Health and our findings support the rationale behind this change.

We also found that mothers preferred to be treated by professionals such as psychologists and family physicians and gave a lower priority for informal professionals such as volunteers and alternative caregivers. These findings are supported by a previous research conducted in Israel showing that about one-third of mothers with PPD symptoms sought treatment from professionals [17].

Our findings showed that women with PPD preferred group interventions and personal psychotherapy, such personal meetings and home visits compared to technology-mediated interventions. This finding is interesting because, as suggested in previous studies, women preferred more personal psychotherapy [20, 24, 25], and were less likely to prefer group interventions [25]. It is possible that group therapy enables mothers to develop better strategies for coping with PPD symptoms by learning from other mothers’ experiences. Group interventions can give mothers a feeling that they are not alone but rather part of a group coping with difficulties of PPD [35]. Women were less likely to prefer technologically mediated interventions, although this type of treatment can reduce the labeling of mental illness and protect their privacy [36]. Future research is warranted to examine this finding. Our findings also reinforce other studies that showed that women are very unlikely to prefer medications for treatment of PPD, which was ranked 12/13 [21, 37, 38].

We found that mothers with PPD compared to mothers without PPD were less likely to seek treatment in general. This finding was supported by previous studies in Israel and abroad [17, 18, 34]. It is possible that mothers with PPD may be afraid that seeking help for their symptoms will be construed as evidence of defective parenting capacity. Thus, the findings of previous studies have shown that fear of tagging as a “bad mother” reduced the reference to treat symptoms of PPD [39, 40]. It is also possible that mothers with PPD symptoms have less energy in general to move outside of their personal realm and initiate help seeking, which reinforces the need for identifying these mothers and bringing treatment to them, through home visits or other means.

It is important to identify the factors that hinder accessing treatment among women with PPD. This behavior raises concerns and emphasizes the need for active reaching out to engage these mothers, to overcome their fear of excess stigmatization in the health and welfare care systems.

The primary limitation of this study is that it is cross-sectional and did not include Arabic-speaking mothers. Future studies should include the attitudes and behavioral intentions of other sectors of Israeli society as well.

## Conclusions

Mothers with PPD represent a high-risk population who can benefit greatly from appropriate treatment. Our findings show that mothers with PPD have unique preferences. Mothers with PPD preferred private mental health practice and community treatment centers by mental healthcare professionals, and preferred group interventions and personal psychotherapy rather than technology-based interventions. We also found that mothers with PPD compared to mothers without PPD were less likely to seek treatment in general.

On the practical level, sharing the findings of this study with professionals, such as nurses at family health centers or physicians, in professional training sessions, lectures, seminars and journals, can increase awareness of their role in providing formal support for mothers who require treatment for PPD.

Our findings may assist professionals to develop relevant policies and programs designed to promote the health of mothers by reaching a deeper understanding of their preferences. As a result, therapy alternatives can be developed to better suit diverse groups of mothers in Israel who cope with the impacts of PPD, for the benefit of mothers, children, and entire families.

## Data Availability

The datasets used and/or analyzed during the current study are available from the corresponding author on reasonable request.

## Appendix 1

**Table 4:** Preferred place of treatment (exploratory factor analysis)

Place of treatment	Community health clinic	Psychiatric clinics	Private mental health practice
MCHCs	.80		
Community health clinic (HMOs)	.69		
Community mental health centers		.72	
Psychiatric hospital outpatient clinic		.84	
General hospital outpatient clinic		.77	
Mental health clinics for women in general hospitals		.73	
Inpatient in a psychiatric hospital		.67	
private mental health practice			.50

## Appendix 2

**Table 5:** Preferred type of professional (exploratory factor analysis)

Type of professional	Professional in the community	Mental health professionals	Allied health workers
Nurse	.54		
Social Worker	.46		
Family Doctor	.60		
Psychiatrist		.69	
Psychologist		.73	
A volunteer trained for this purpose			.66
Occupational therapist			.68
Alternative therapist			.66
Art/ Movement/ Music therapist			.71

## Appendix 3

**Table 6:** Preferred mode of treatment (exploratory factor analysis)

Mode of treatment	Personal based interventions	Technology mediated interventions	Group based interventions
Home visits	.49		
Personal meetings in the community clinic	.44		
Personal meetings in a private office	.55		
Medications	.62		
Medications and treatment	.72		
Treatment by phone		.60	
Skype with a professional		.75	
Internet-based treatment, chatting with a professional		.77	
Internet-based treatment, chatting with another woman in my situation		.70	
Group meetings on the web with women with the same problems as a self-help group		.74	
Practice on a computer site with help and explanation of therapist, online		.66	
Using an iPhone application		.62	
Group meetings in the community under the guidance of a professional			.75
Group meetings community under the guidance of a woman who is not a professional but has experienced similar problems			.72

## Abbreviations

EFA: Exploratory Factor Analysis
HMOs: Health Maintenance Organizations
MCHCs: Maternal Child Health Clinics
PPD: Post-partum depression

## References

[1] American Psychiatric Association. Diagnostic and statistical manual of mental disorders: DSM–5. American Psychiatric Pub. 2013.

[2] Robertson Emma, Grace Sherry, Wallington Tamara, Stewart Donna E (2004). Antenatal risk factors for postpartum depression: a synthesis of recent literature. General Hospital Psychiatry.

[3] Yelland J, Sutherland G, Brown SJ. Postpartum anxiety, depression and social health: findings from a population-based survey of Australian women. BMC Public Health. 2010;10(1):771. 10.1186/1471-2458-10-771 PMC3022850 21167078

[4] Reck C., Struben K., Backenstrass M., Stefenelli U., Reinig K., Fuchs T., Sohn C., Mundt C. (2008). Prevalence, onset and comorbidity of postpartum anxiety and depressive disorders. Acta Psychiatrica Scandinavica.

[5] Wisner Katherine L., Chambers Christina, Sit Dorothy K. Y. (2006). Postpartum Depression. JAMA.

[6] Wisner Katherine L., Sit Dorothy K. Y., McShea Mary C., Rizzo David M., Zoretich Rebecca A., Hughes Carolyn L., Eng Heather F., Luther James F., Wisniewski Stephen R., Costantino Michelle L., Confer Andrea L., Moses-Kolko Eydie L., Famy Christopher S., Hanusa Barbara H. (2013). Onset Timing, Thoughts of Self-harm, and Diagnoses in Postpartum Women With Screen-Positive Depression Findings. JAMA Psychiatry.

[7] Glasser S., Barell V., Boyko V., Ziv A., Lusky A., Shoham A., Hart S. (2000). Postpartum depression in an Israeli cohort: Demographic, psychosocial and medical risk if actors. Journal of Psychosomatic Obstetrics & Gynecology.

[8] Glasser Saralee, Stoski Ela, Kneler Victoria, Magnezi Racheli (2011). Postpartum depression among Israeli Bedouin women. Archives of Women's Mental Health.

[9] Dennis C.-L. E., Janssen P. A., Singer J. (2004). Identifying women at-risk for postpartum depression in the immediate postpartum period. Acta Psychiatrica Scandinavica.

[10] Akman I (2006). Mothers' postpartum psychological adjustment and infantile colic. Archives of Disease in Childhood.

[11] Patel V (2003). Postnatal depression and infant growth and development in low income countries: a cohort study from Goa, India. Archives of Disease in Childhood.

[12] Diego Miguel A., Field Tiffany, Hernandez-Reif Maria, Cullen Christie, Schanberg Saul, Kuhn Cynthia (2004). Prepartum, Postpartum, and Chronic Depression Effects on Newborns. Psychiatry: Interpersonal and Biological Processes.

[13] Goodman Janice H. (2004). Paternal postpartum depression, its relationship to maternal postpartum depression, and implications for family health. Journal of Advanced Nursing.

[14] Gjerdingen D. K., Yawn B. P. (2007). Postpartum Depression Screening: Importance, Methods, Barriers, and Recommendations for Practice. The Journal of the American Board of Family Medicine.

[15] Ministry of Health. Procedure for Locating Women at Risk for Depression in Pregnancy and Postpartum, Public Health Services, No. 20/12. 2014.

[16] Cox JL, Holden JM (2003). Perinatal mental health: a guide to the Edinburgh postnatal depression scale (EPDS).

[17] Bina Rena (2014). Seeking Help for Postpartum Depression in the Israeli Jewish Orthodox Community: Factors Associated with Use of Professional and Informal Help. Women & Health.

[18] Horowitz June Andrews, Cousins Ann (2006). Postpartum Depression Treatment Rates for At-Risk Women. Nursing Research.

[19] Forder PM, Rich J, Harris S, Chojenta C, Reilly N, Austin MP, et al. Honesty and comfort levels in mothers when screened for perinatal depression and anxiety. Women and Birth. 2019. 10.1016/j.wombi.2019.04.001 31133524

[20] Cooper Peter J., Murray Lynne, Wilson Anji, Romaniuk Helena (2003). Controlled trial of the short- and long-term effect of psychological treatment of post-partum depression. British Journal of Psychiatry.

[21] O'Hara Michael W., Stuart Scott, Gorman Laura L., Wenzel Amy (2000). Efficacy of Interpersonal Psychotherapy for Postpartum Depression. Archives of General Psychiatry.

[22] Milgrom J, Holt CJ, Gemmill AW, Ericksen J, Leigh B, Buist A, et al. Treating postnatal depressive symptoms in primary care: a randomised controlled trial of GP management, with and without adjunctive counselling. BMC Psychiatry. 2011;11(1):95. 10.1186/1471-244X-11-95 PMC3121669 21615968

[23] McGarry Joanne, Kim Han, Sheng Xiaoming, Egger Marlene, Baksh Laurie (2009). Postpartum Depression and Help-Seeking Behavior. Journal of Midwifery & Women's Health.

[24] O'Mahen Heather A., Flynn Heather A. (2008). Preferences and Perceived Barriers to Treatment for Depression during the Perinatal Period. Journal of Women's Health.

[25] Goodman Janice H. (2009). Women’s Attitudes, Preferences, and Perceived Barriers to Treatment for Perinatal Depression. Birth.

[26] Munoz Christina, Agruss Janyce, Haeger Amy, Sivertsen Lynn (2006). Postpartum Depression: Detection and Treatment in the Primary Care Setting. The Journal for Nurse Practitioners.

[27] Glasser S, Barell V. Depression scale for research in and identification of postpartum depression. Harefuah. 1999;136(10):764–8. 10955108

[28] Jimenez Daniel E., Bartels Stephen J., Cardenas Veronica, Dhaliwal Sanam S., Alegría Margarita (2012). Cultural Beliefs and Mental Health Treatment Preferences of Ethnically Diverse Older Adult Consumers in Primary Care. The American Journal of Geriatric Psychiatry.

[29] Jardri Renaud, Pelta Jerome, Maron Michel, Thomas Pierre, Delion Pierre, Codaccioni Xavier, Goudemand Michel (2006). Predictive validation study of the Edinburgh Postnatal Depression Scale in the first week after delivery and risk analysis for postnatal depression. Journal of Affective Disorders.

[30] Johnstone Stuart J., Boyce Philip M., Hickey Anthea R., Morris-Yates Allen D., Harris Meredith G. (2001). Obstetric Risk Factors for Postnatal Depression in Urban and Rural Community Samples. Australian & New Zealand Journal of Psychiatry.

[31] Green Katherine, Broome Hazel, Mirabella James (2006). Postnatal depression among mothers in the United Arab Emirates: Socio-cultural and physical factors. Psychology, Health & Medicine.

[32] Forman DR, O’Hara MW, Stuart S, Gorman LL, Larsen KE, Coy KC. Effective treatment for postpartum depression is not sufficient to improve the developing mother-child relationship. Dev Psychopathol. 2007;19(2):585–602. 10.1017/S0954579407070289 17459185

[33] Eilat-Tsanani S, Merom A, Romano S, Reshef A, Lavi I, Tabenkin H. The effect of postpartum depression on womens’ consultations with physicians. Isr Med Assoc J. 2006;8(6):406. 16833170

[34] Evins GG, Theofrastous JP, Galvin SL. Postpartum depression: a comparison of screening and routine clinical evaluation. Am J Obstet Gynecol. 2000;182(5):1080–2. 10.1067/mob.2000.105409 10819833

[35] Scope A, Leaviss J, Kaltenthaler E, Parry G, Sutcliffe P, Bradburn M, et al. Is group cognitive behaviour therapy for postnatal depression evidence-based practice? A systematic review. BMC Psychiatry. 2013;13(1):321. 10.1186/1471-244X-13-321 PMC4219505 24283266

[36] O'Mahen Heather A., Woodford Joanne, McGinley Julia, Warren Fiona C., Richards David A, Lynch Thomas R., Taylor Rod S. (2013). Internet-based behavioral activation—Treatment for postnatal depression (Netmums): A randomized controlled trial. Journal of Affective Disorders.

[37] Brandon Anna R., Freeman Marlene P. (2011). When She Says “No” to Medication: Psychotherapy for Antepartum Depression. Current Psychiatry Reports.

[38] Pearlstein T. B., Zlotnick C., Battle C. L., Stuart S., O’Hara M. W., Price A. B., Grause M. A., Howard M. (2006). Patient choice of treatment for postpartum depression: a pilot study. Archives of Women's Mental Health.

[39] Wisner Katherine L., Perel James M., Peindl Kathleen S., Hanusa Barbara H., Piontek Catherine M., Findling Robert L. (2004). Prevention of Postpartum Depression: A Pilot Randomized Clinical Trial. American Journal of Psychiatry.

[40] Wisner Katherine L., Hudson Scholle Sarah, Stein Bradley (2008). Perinatal Disorders: Advancing Public Health Opportunities. The Journal of Clinical Psychiatry.

